# The Endothelium as a Hub for Cellular Communication in Atherogenesis: Is There Directionality to the Message?

**DOI:** 10.3389/fcvm.2022.888390

**Published:** 2022-04-15

**Authors:** Kathryn L. Howe, Myron Cybulsky, Jason E. Fish

**Affiliations:** ^1^Toronto General Hospital Research Institute, University Health Network, Toronto, ON, Canada; ^2^Institute of Medical Science, University of Toronto, Toronto, ON, Canada; ^3^Peter Munk Cardiac Centre, University Health Network, Toronto, ON, Canada; ^4^Division of Vascular Surgery, Department of Surgery, University of Toronto, Toronto, ON, Canada; ^5^Department of Laboratory Medicine and Pathobiology, University of Toronto, Toronto, ON, Canada

**Keywords:** endothelium, crosstalk, extracellular vesicles, atherosclerosis, polarity, directionality, inflammation, microRNA

## Abstract

Endothelial cells line every blood vessel and thereby serve as an interface between the blood and the vessel wall. They have critical functions for maintaining homeostasis and orchestrating vascular pathogenesis. Atherosclerosis is a chronic disease where cholesterol and inflammatory cells accumulate in the artery wall below the endothelial layer and ultimately form plaques that can either progress to occlude the lumen or rupture with thromboembolic consequences – common outcomes being myocardial infarction and stroke. Cellular communication lies at the core of this process. In this review, we discuss traditional (e.g., cytokines, chemokines, nitric oxide) and novel (e.g., extracellular vesicles) modes of endothelial communication with other endothelial cells as well as circulating and vessel wall cells, including monocytes, macrophages, neutrophils, vascular smooth muscle cells and other immune cells, in the context of atherosclerosis. More recently, the growing appreciation of endothelial cell plasticity during atherogenesis suggests that communication strategies are not static. Here, emerging data on transcriptomics in cells during the development of atherosclerosis are considered in the context of how this might inform altered cell-cell communication. Given the unique position of the endothelium as a boundary layer that is activated in regions overlying vascular inflammation and atherosclerotic plaque, there is a potential to exploit the unique features of this group of cells to deliver therapeutics that target the cellular crosstalk at the core of atherosclerotic disease. Data are discussed supporting this concept, as well as inherent pitfalls. Finally, we briefly review the literature for other regions of the body (e.g., gut epithelium) where cells similarly exist as a boundary layer but provide discrete messages to each compartment to govern homeostasis and disease. In this light, the potential for endothelial cells to communicate in a directional manner is explored, along with the implications of this concept – from fundamental experimental design to biomarker potential and therapeutic targets.

## Introduction: Endothelial Cells as Gatekeepers

**Figure 1:**
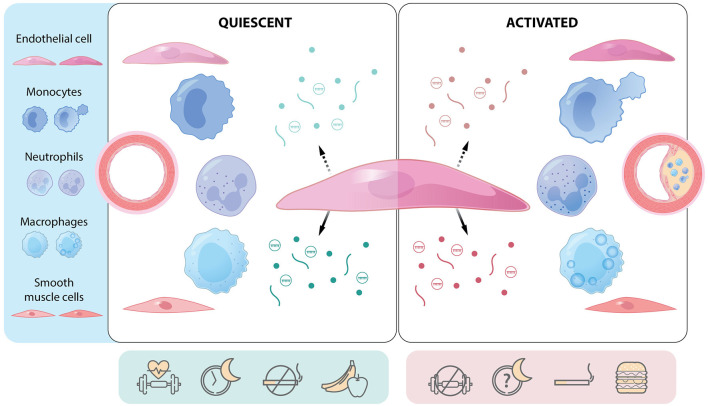
The endothelial cell as a communication hub in quiescent and activated states. Central endothelial cell with apical (hatched arrow) and basolateral (solid arrow) release of mediators [e.g., proteins (lines), gases and small molecules (dots), extracellular vesicles (circles containing cargo)]. Communication differs depending on whether the endothelium is quiescent or activated (e.g., regions prone to atherosclerosis) and can affect several cell types. Endothelial function and communication are affected by several lifestyle factors such as physical activity, sleep hygiene, smoking, and diet. Atherosclerotic plaque development is a function of cellular communication and represents a multicellular response to environmental, genetic, and epigenetic cues. Green: mediators and factors that actively maintain endothelial quiescence. Red: mediators and factors that promote endothelial activation.

Endothelial cells (ECs) lie at a critical interface between the circulating blood and cellular milieu below, and hence serve as gatekeepers of vascular biology (1). Lining every blood vessel throughout the human body, this linked network of cells performs key roles unique to the local environment to maintain organ homeostasis. In large and medium-sized arteries, endothelial cells function as an anti-thrombotic physiological barrier, are sensors of mechanical forces, oxygen tension and nutrient availability, serve as sentinels of infectious threats, and mount appropriate responses to distortions in normal physiology to ensure tissue viability (2). Intercellular communication is at the core of this homeostatic process. Through soluble mediators such as nitric oxide and other small molecules, release of growth factors and cytokines, production or degradation of extracellular matrix, and secretion of extracellular vesicles (EVs) containing nucleic acids, proteins, and lipids, endothelial cells can govern one another, nearby cells in the local milieu, and cells located in remote tissues (1, 3, 4). Control of permeability (e.g., glycocalyx and junctional proteins), transcytosis (e.g., transcellular transport), and basement membrane composition, are additional signaling hubs where endothelial cells govern communication between the blood and tissue (5–7). Completing this communication loop, the endothelium is also exquisitely sensitive to mechanical and biochemical signals in their microenvironment. Regardless of whether an EC communication strategy is just being uncovered (e.g., EVs) or has been investigated for decades (e.g., nitric oxide), they each play a critical role in conditions such as atherosclerosis where vascular homeostasis is dysregulated. In the following sections, the nature of endothelial communication – both autocrine and paracrine – is explored, with a specific focus on novel modes of communication. Additionally, we will discuss the impact of cellular plasticity on cell communication and will consider approaches to exploit these lines of communication therapeutically. Finally, we will determine whether there is evidence for directionality of the messages that are sent and received (Figure 1). Together, this review will provide the reader with a highly focused update on the dynamic role of the endothelium as a maestro in cellular communication in atherosclerosis and provide provocative insights into how these signals might be intercepted or modulated to mitigate disease.

## Role of the Endothelium and Cell-Cell Crosstalk in Health and Atherosclerosis

In its healthy state, the endothelium is quiescent and autocrine and paracrine communication emanating from ECs preserves organ function [reviewed in (8)]. Endothelial quiescence is a process that must be actively maintained – it is not a “default” setting. As Ricard et al. elegantly discuss in their review (8), single cell sequencing is highlighting the regional- and organ-specific heterogeneity of ECs (i.e., lymphatic vs. venous vs. arterial and large-vessel vs. capillary) (9) and is uncovering the distinct signaling pathways required for EC quiescence. For example, in the vasculature, endothelial barrier function and cell fate is maintained by the Angiopoietin/Tie2 pathway and limitation of TGF-β signaling, respectively, among others (10, 11). The importance of preserving endothelial quiescence has been well-illustrated: loss of endothelial nitric oxide synthase accelerates murine atherosclerosis and aortic aneurysm formation (12), while endothelial-specific *MAPK1/2* (ERK1/2) knockout in mice has a catastrophic impact, with hypertension, decreased endothelial nitric oxide synthase expression, increased endothelin-1 expression, and death within 5 weeks (13). Bi-directional cellular communication also participates in endothelial homeostasis. Beyond autocrine signals from the endothelium, monocytes patrolling the apical surface of the endothelium have been shown to protect against endothelial death (i.e., apoptosis) and limit inflammation – with a large artery model suggesting these cells possibly serve a housekeeping function to preserve endothelial integrity in the face of hyperlipidemia and atherosclerosis (14). Conversely, loss of endothelial quiescence causes a shift in metabolism and loss of critical protective communication via soluble mediators, including nitric oxide (15, 16). Equally important to endothelial homeostasis is physiologic high shear stress from laminar blood flow, modulated in part by the anti-inflammatory and cytoprotective actions of the transcription factor, Kruppel-like factor 2. Reduced or oscillatory shear stress, as found in arterial branch points and curvatures, renders the artery prone to atherosclerotic plaque development. Recent systems biology and omics approaches are revealing that endothelial responses to low shear stress involves activation of developmental pathways such as WNT, Notch, HIF1-α, and Hippo-YAP-Taz [reviewed in (17)] – consequently, these once dormant embryonic programs lead to altered endothelial signaling and undoubtedly contribute to the altered EC communication prevalent in atherosclerotic plaques.

Atherosclerosis is a disease of aging and cellular senescence (18). Atherosclerotic plaques are a complex collection of lipids, extracellular matrix, cells, and cellular debris that accumulate in the vessel wall (19). Plaque formation has a predilection for vascular branch points, where ECs are exposed to disturbed laminar blood flow, resulting in activated endothelial phenotypes (20). Activation of the endothelium can include the following core changes: (1) upregulation of proinflammatory cytokines (e.g., IL-1β, TNF-α) and chemokine production; (2) expression of adhesion molecules such as VCAM-1 and ICAM-1, which are critical for the recruitment of circulating immune cells; (3) loss of an anti-thrombotic surface; (4) upregulation of class II HLA molecules with consequent function in antigen presentation; and (5), loss of barrier integrity (21). The dysfunctional vascular endothelium as a driver of atherogenesis has been recently reviewed (22). In contrast to classic dogma citing loss of endothelial barrier integrity and passive movement of low-density lipoprotein (LDL) into the vessel wall (23, 24), transport of LDL cholesterol (a critical event in atherogenesis) requires active endothelial transcellular transport via expression of scavenger receptor class B type 1 (SR-B1) (25–27) and potentially other molecules such as activin-like kinase 1 (28). In regions of endothelial activation, cellular communication mediated by expression of adhesion molecules and chemokines leads to recruitment of inflammatory cells, including neutrophils (29) and monocytes (30), and accumulation and maintenance of macrophage populations through survival/proliferation signals (31, 32). New data is emerging however, that beyond the prototypic adhesion molecules, ECs have the intrinsic capacity to form “hotspots” for transendothelial neutrophil migration through the formation of junctional membrane protrusions in regions of high Rac1 activity (33). The process is asymmetric within the endothelial luminal and abluminal surfaces and heterogeneous between ECs, with more comprehensive mechanistic understanding still being unraveled (34). While beyond the scope of this review, the reader is referred to work from the Muller laboratory delineating the role of the lateral border recycling compartment – a key region communicating transmigration of leukocytes across ECs (35).

While there are well-described roles for endothelial-monocyte/macrophage communication through cell adhesion molecules, cytokines and chemokines in atherosclerotic plaques (36), more recently, it has been shown that local, but not systemic, production of colony-stimulating factor 1 from endothelial and smooth muscle cells provide macrophage survival signals that drive macrophage proliferation and atherosclerotic plaque progression (37). Atherosclerotic plaque progression is a complex process and develops in response to local and systemic intercellular communication. For many diseases – atherosclerosis included – emerging studies have focused on nanoparticle sized packages of information (EVs) and how intercellular communication is regulated through secretion and uptake of EVs and their contents (4). Endothelial-derived EV communication is beginning to be understood in the context of models of atherosclerosis (38). EVs are heterogeneous and include exosomes, derived from the multivesicular body, and microparticles, derived from budding and excision of cell membrane. EV cargo contains a range of biologically relevant material, including lipids, nucleic acids (e.g., microRNA), and proteins. Of these, microRNA has been studied extensively. These short sequences of non-coding RNA bind to the 3′ untranslated regions of mRNAs via their seed sequences and destabilize mRNAs or repress their translation into protein. Each microRNA typically has multiple mRNA targets, and thus microRNAs are considered as microregulators of health and disease (39, 40). The microRNA content of EVs has begun to be explored in the context of atherogenesis, and comprehensive reviews of their role in atherosclerosis (41) and carotid artery atherosclerotic disease (42) are available. This form of cellular crosstalk is also beginning to explain gaps in our understanding of acute myocardial infarction pathogenesis. For example, VCAM-1 positive endothelial-derived EVs enriched in microRNA-126 are responsible for the rapid neutrophil mobilization from the spleen, making these EVs a potential therapeutic target to limit infarct size (43).

*In vitro* models are the simplest way to examine EV communication. This reductionist approach has revealed that EVs derived from quiescent ECs have different effects on cells than those derived from inflamed (e.g., TNF-α stimulated) ECs (44). For example, under healthy quiescent conditions, ECs suppress monocyte activation by release of EVs containing anti-inflammatory microRNAs (45, 46). Similarly, ECs can communicate atheroprotective microRNAs to vascular smooth muscle cells via EVs (47). When ECs are activated with pro-inflammatory cytokines or oscillatory shear stress, they release EVs containing miR-92a, which cause macrophage activation, increased lipoprotein uptake, and decreased migration (48). In response to stress from serum-starvation, ECs release EVs containing the inflammatory adhesion molecule VCAM-1, which drive inflammatory and senescent pathways in vascular smooth muscle cells (49). Although *in vitro* studies suggest that endothelial-derived EVs may serve as an important mode of intercellular communication, they lack the complexity of animal models. To that end, reporter systems in mice and zebrafish may provide new opportunities to track endothelial-derived EVs and better evaluate intercellular communication in complex disease phenotypes such as atherosclerosis (50–52).

## Endothelial Plasticity in Atherosclerosis: Altered Cell-Cell Communication?

Cellular plasticity and diversity within atherosclerotic plaques has been increasingly appreciated in recent years (53). Single cell RNA sequencing is a robust tool that has revealed in intricate detail the complexity of the atherosclerotic plaques [reviewed in (54)]. Lineage-tracing studies are supporting the notion that cellular identities are on a continuum and have the potential to “transform”, where cells acquire the phenotype and properties typically attributed to another cell [e.g., macrophage subsets (55, 56) and vascular smooth muscle cell plasticity and plaque development (57, 58)]. Notably, ECs can undergo partial or complete endothelial-to-mesenchymal transition (EndMT), where classic cell-cell junction proteins are lost, and mesenchymal markers are gained. This is often, but not always, associated with enhanced migratory capabilities and delamination from the endothelial monolayer, allowing cells to migrate into the plaque. Ligand-receptor interactions from transcriptomics are likewise informing our understanding of cellular communication in states of health and disease in the cardiovascular system (59). The Giannarelli laboratory studied the immune landscape of human carotid atherosclerotic plaques and found distinct T cell and macrophage populations that differed from the blood and importantly, the putative cell-cell interactions occurring in plaques from patients that had clinical cerebrovascular events (i.e., strokes) (60). The Lutgens laboratory observed the diversity of the endothelium overlying atherosclerotic plaques in both mice and humans, with electron microscopic images showing greater abnormalities in early plaques and at the shoulder regions (61). The junctional disruptions and large transcellular endothelial pores seen in early plaques and aggravated on the shoulders of advanced plaques are very likely to influence communication between the blood and the medial layer of the vessel wall (61). Moreover, these locations for endothelial transformations may correspond to the extensive proatherogenic endothelial reprogramming in response to disturbed flow that has been revealed by single-cell RNA and chromatin accessibility studies (62). Indeed, Andueza et al. show EC responses to disturbed flow are highly plastic, with evidence that disturbed laminar flow leads to a transition to pro-inflammatory cells, endothelial progenitor cells, EndMT, and even a novel immune-like cell (EndICLT) (62). While lineage tracing studies will be needed to confirm the origin of EndICLT cells, prior lineage tracing studies have shown that EndMT occurs during atherosclerotic plaque development in mice (63). Moreover, analysis of human tissue has shown that EndMT correlates with an unstable plaque phenotype, which is likely driven by altered collagen and matrix metalloproteinase expression causing decreased cap thickness (63). However, EndMT also contributes to formation of the fibrous cap and may therefore have protective, as well as detrimental roles (64). As TGF-β is a key driver of EndMT, there is work showing that limiting endothelial responses to TGF-β (i.e., by endothelial-specific *Tgfbr1/2* knock out in mouse models of atherosclerosis) limits EndMT, decreases inflammation and plaque progression, and even facilitates plaque regression (65). However, TGF-β is also a potent anti-inflammatory cytokine, and a more nuanced therapeutic target will therefore be required (66). What is clear, however, is that endothelial activation and cellular plasticity are drivers of atherosclerotic disease. Whether this cell identity shift is truly “fluid” and reversible is unknown – it is certainly conceivable, given the observation that mesenchymal to epithelial transition occurs in development and reprogramming (67). Regardless, given ongoing cell-cell communication within the atherosclerotic plaque environment, ECs transitioning to other functional cell phenotypes will undoubtedly send and receive different messages to/from their neighboring cells. As seen in *in vitro* models, ECs induced to undergo EndMT produce a different EV cargo that affect the metabolism and angiogenic potential of naïve recipient ECs (68). We must now look at more complex tissue to understand how altered endothelial signals can be harnessed to detect disease earlier, intervene, and/or identify targets for plaque stabilization or regression. To that end, Depuydt et al. performed single cell transcriptomics and chromatin accessibility on human carotid atherosclerotic plaques and not only identified 4 distinct endothelial populations, but also utilized ligand-receptor interactions between cell types to predict endothelial communication with myeloid and smooth muscle populations (53). Together, these studies make the case for continued work to focus on EC plasticity and consequent EC-myeloid and EC-smooth muscle cell communication in atherosclerotic disease. Moreover, there is emerging evidence that EVs act on ECs to govern cell fate/plasticity – this will undoubtedly affect the communication loop between the endothelium in both a local and systemic manner (68, 69).

## Directionality of Endothelial Communication – a Provocative Idea With Implications for Cell-Cell Crosstalk?

**Figure 2:**
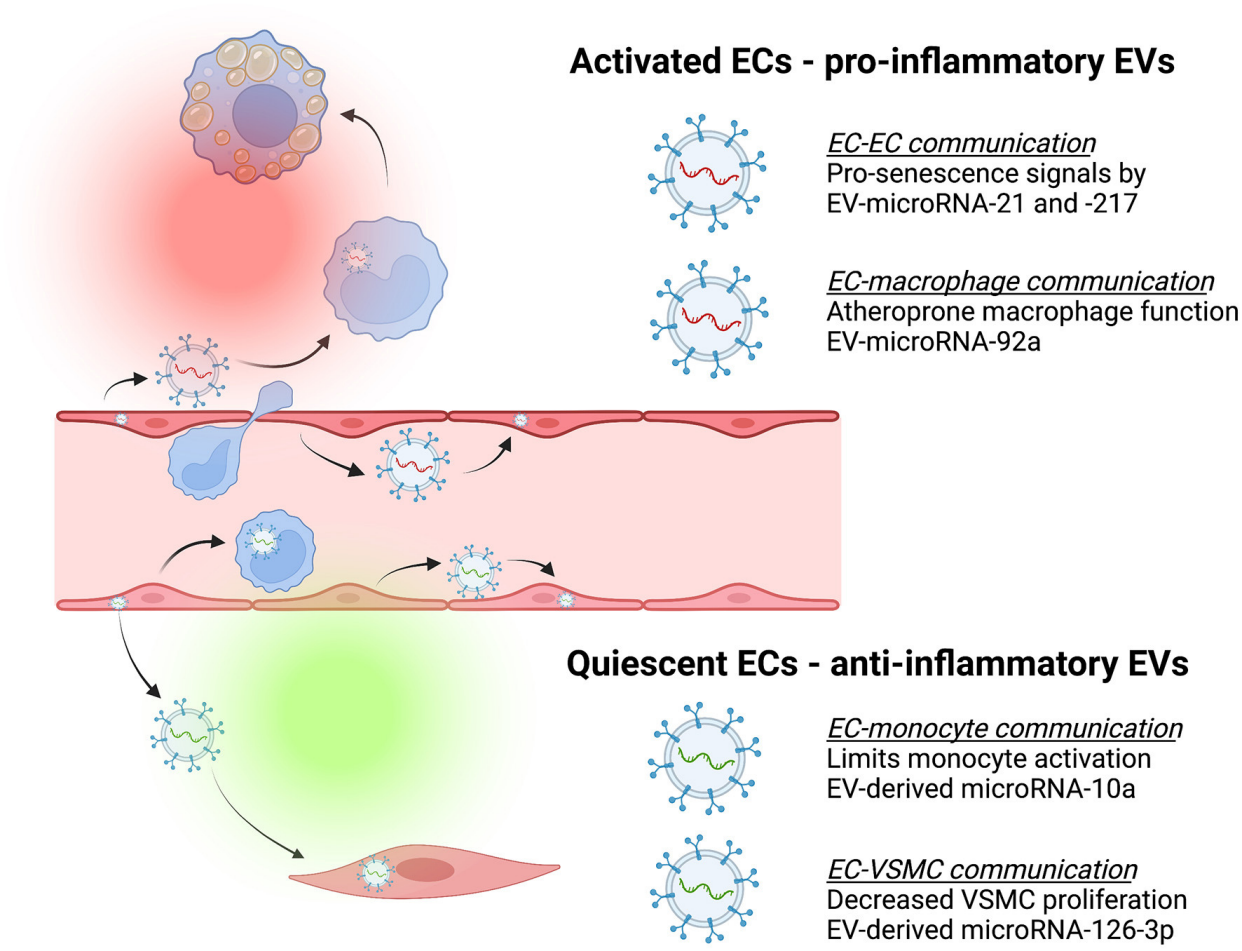
Endothelial-derived extracellular vesicles (EVs) contribute to cell-cell communication that can be atheroprotective (green) or atheroprone (red). Endothelial cells (ECs) release EVs containing different cargo depending on whether they are activated or quiescent, and these have effects on recipient cells [e.g., monocytes, macrophages, and vascular smooth muscle cells (VSMC)]. For example, activated ECs release EVs that deliver pro-senescence signals such as microRNA-21 and microRNA-217 to other ECs (78) or separately, release EVs containing microRNA-92a capable of inducing macrophage inflammation and impaired migration (48). In contrast, quiescent ECs release EVs containing cargo such as microRNA-10a that limit monocyte activation (45) or EVs containing microRNA-126-3p to decrease VSMC proliferation and neointima formation (79).

Cellular polarity is common across tissues, particularly for cells that serve as boundary layers. In the vasculature, endothelial cells have apical-basal polarity to establish a barrier between the circulation (i.e., blood) and tissues (70). Key functions are performed by each surface, with polarized expression of anticoagulant factors, receptors, ion channels, mechanosensors, and junctional proteins conferring regional properties (71). Importantly, uniform laminar and disturbed laminar blood flow have distinct spatio-temporal shear stress profiles and impart unique frictional forces that are sensed by ECs through multiple mechanosensors, including Peizo1 (72, 73). Endothelial mechanosensing contributes to the regulation of many aspects of EC biology, including shape, orientation, and polarity. Recent work has shown that PAR3 expression by ECs governs endothelial polarity relative to the direction of blood flow but not apical-basal polarity (74). Conversely, Scrib is a polarity protein that helps to maintain apical-basolateral polarity, endothelial quiescence, and confers atheroprotection (75). Notably, the endothelial secretome is also polarized, with proteomic analysis showing that extracellular matrix components are predominantly secreted basally while the apical proteome contains a significant proportion of EV proteins (76). Further, ECs respond to inflammatory stimuli (TNF-α) differentially depending on whether the apical vs. basolateral surface is exposed (77). Endothelial-derived EVs contribute to the circulating secretome and carry cargo capable of governing endothelial function in a paracrine and perhaps endocrine manner, as well as other cells: truly, EC-EVs have been referred to as “keepers of health” as well as “messengers of disease” (50). As ECs are situated at the interface of the blood vessel lumen and wall, it is conceivable they selectively load and directionally release EVs containing different cargo depending on the stimulus (Figure 2). Other cells in similar physiological environments, such as retinal pigmented epithelium and intestinal epithelial cells, demonstrate polarized release of exosomes containing differential protein content (80, 81). Support for the concept that ECs communicate with directional particle release is further supported by Yun et al. who showed human brain microvascular endothelial cells release microparticles (microvesicles) constitutively and in response to pro-inflammatory cytokines and critically, that protein content and functional effects (permeability) were different between apical and basolateral sources (82). Given the unique location of ECs, communication from activated cells might reflect critical loco-regional information and this information may be shared through distinct messages via apical and basolateral EV release – providing unique candidates for diagnostic and therapeutic targets to identify at-risk patients and intervene at different stages of atherosclerotic plaque development.

## The Endothelium as a Therapeutic Target in Atherosclerosis: can we Exploit EV-Based Cell-Cell Crosstalk?

It is clear the endothelium actively governs cardiovascular health. From pro-homeostatic signals such as nitric oxide release (1, 15), to atheroprotective EVs (45, 47), to molecules that facilitate resolution of inflammation (83–85), there are several means by which endothelial cross-talk is beneficial. As the endothelium becomes activated with transition to distinct phenotypes, cellular communication is altered, such that these ECs now have the capacity to mediate atherosclerotic disease through cell-cell crosstalk. Strategies should therefore be designed to preserve or “boost” homeostatic roles of the endothelium or alternatively, target dysregulated endothelium to modulate atherosclerotic plaque development. The concept that EVs participate in intercellular communication is firmly established and multiple groups are exploring their diagnostic and therapeutic potential. As these lipid bilayers are ubiquitously released throughout the body, protect their molecular cargo from degradation, carry surface markers identifying the parent cell, and can be employed as delivery systems, they have tremendous potential in multiple disease applications (86, 87). If we consider the profound significance of the endothelium located at the interface of the vessel wall where it participates in systemic (i.e., blood) and local (i.e., vessel wall) communication, it may be that modifying endothelial function is a viable target in cardiovascular disease. Here, we consider a few novel endothelial-specific approaches in the context of EV communication and atherosclerosis: (1) designing treatments that preserve endothelial quiescence and limit senescence; (2) interrupting EC communication promoting early atherosclerotic plaques; and (3), selectively targeting regions of activated endothelium to deliver plaque-stabilizing/regression therapies to the atherosclerotic plaque lying below.

### Preserving Endothelial Quiescence Through EV-Mediated Delivery of Bioactive Molecules

Identifying EV components that govern endothelial health may be difficult as few studies have focused on this process. However, we can look to recent work on the protective effects of a healthy lifestyle on atherosclerotic disease, such as nutrition, sleep, stress reduction and exercise, to provide some clues on where to start (88). To date, a prototypic functional measure of endothelial health is flow-mediated vasodilatation (FMD). Human studies exploring Mediterranean diets have demonstrated the benefits on EC function when assessed by FMD (89). More recently, the benefits of carbohydrate restriction improved FMD, markers of endothelial activation, and decreased endothelial microparticles in subjects with type 2 diabetes (90). Sleep is also critical for health. Key research by the Swirski laboratory has emerged showcasing the importance of sleep quality in the control of hematopoiesis and atheroprotection (91). Endothelial health is specifically affected by altered circadian rhythms and sleep deprivation, with reduced FMD observed by several investigators (92, 93). Notably, aerobic exercise is considered a particularly effective counterstrategy through its ability to limit endothelial inflammation and bolster nitric oxide production (94). Clearly, exercise affects multiple cellular populations, with cardiovascular health depending upon orchestrated crosstalk between them (95). A comprehensive evaluation by Brahmer et al. has demonstrated leukocyte, platelet, and endothelial-derived EVs increase in response to exercise and represent a complex communication network that could be exploited to preserve endothelial health (96). This concept of “exerkines” has been recently established in models of cardioprotection, which showed that both exercise training or laminar shear stress directly enhanced EV-microRNA-342-5p in ECs to provide cardiomyocyte protection (97). Whether or not there is a similar beneficial autocrine effect on ECs is unknown (97). As we seek to understand how cardiovascular fitness can be preserved through maintenance of endothelial health and potentially EV-based therapeutics, it would be prudent to discern whether novel mediators act on ECs alongside other cellular targets. In this light, some of the most promising work might evolve from studies determining EV cargo produced by senescent ECs – key insights precisely because of the paracrine induction of senescence on younger cells through a “bystander effect” from aged cells (98). Reversing or preventing effectors of senescence would thereby limit “spread” and preserve endothelial health. Indeed, recent work by Mensa et al. determined that senescent endothelial EVs spread pro-senescence signals through microRNA-21 and−217, affecting DNA methylation and cell replication, and these have an age-specific human biomarker correlate when studied across subjects 40–100 years of age (78). The therapeutic potential of using EVs to program target cells with anti-senescence or rejuvenation strategies with a range of natural EV sources (e.g., young donor-derived) and artificial (e.g., nanomedicines) is an area of active exploration and promise (99). Taken together, there are multiple early insights that can be further explored from work being done on circadian rhythms, exercise, nutrition and aging to target endothelial health and prevent atherosclerosis.

### Interrupting Early Atherosclerosis

An additional target to limit atherosclerotic disease would be honing endothelial-directed strategies to interrupt early plaque development. The accumulation of LDL in the subendothelial space would be a reasonable target. Antagonism of endothelial SR-B1 (25–27) or downstream regulators of LDL transcytosis and diverting LDL transcytosis to endolysosomal degradation (100) could be promising approaches, but will require teasing apart the pathways used by SR-B1 to effect the anti-atherogenic process of reverse cholesterol transport (6). Alternatively, immunotargeting activated ECs provides a strategy for delivering therapies to atheroprone regions. While not new (101), this concept has renewed potential in the era of EVs and nanomedicines. Recent work by Distasio and colleagues demonstrated VCAM-1-targeted gene delivery of nanoparticles (NPs) containing the anti-inflammatory cytokine interleukin-10, localized to inflamed ECs and atherosclerotic plaques (102). The Fang laboratory and others have done elegant work over the past decade to not only identify that disturbed flow-induced microRNA-92a causes endothelial dysfunction (103, 104), but also recently reported VCAM-1 targeted NPs can preferentially deliver microRNA-92a inhibitors to inflamed ECs and reduce aortic atherosclerotic plaque development in a murine model (105). Considering endogenous sources, there is certainly promise that endothelial-derived EVs play roles in the progression of atherosclerosis (106); by focusing on those that are considered beneficial such as microRNA-10a (45) or microRNA-126 (79, 107) and what drives their expression, we may likewise find strategies to interrupt early atherogenesis. Early work from the Fish laboratory revealed that microRNA-146 repressed endothelial activation (108), with later *in vivo* work showing its importance in restraining EC activation and atherosclerosis (109). As highlighted, targeting the activated endothelium with site-directed approaches to limit atherogenesis has some clear promise – at least in pre-clinical models. Selectively targeting advanced atherosclerotic plaques will have different, but perhaps more clinically relevant, objectives; namely, to stabilize and protect from rupture.

### Selective Targeting Over Advanced Plaque

The advanced atherosclerotic plaque has unique biology with distinct therapeutic targets that are emerging. One heavily studied target is microRNA-33 (110, 111). Studies using non-human primates have demonstrated that pharmacological inhibition of microRNA-33 can favorably improve cholesterol profiles, with the potential for reducing cardiovascular disease (112). Further insights from the Moore laboratory have shown that microRNA-33 silencing reprograms the immune cell landscape in atherosclerotic plaques to promote regression (113). Intriguingly, ECs might serve as a “trojan horse” for anti-microRNA-33 therapy: using adenoviral delivery, ECs can be steered to release exosomes containing anti-microRNA-33a-5p that transfer their contents to macrophages and vascular smooth muscle cells to enhance cholesterol efflux (114). Beyond the microRNA-33 family, additional novel targets exist within resolution biology pathways. Defective efferocytosis (clearing of the dead cells) has been associated with advanced atherosclerotic plaques and is considered the linchpin in plaque vulnerability (115). EVs from cardiac progenitor-derived cells enhance macrophage efferocytosis through microRNA-26 to sustain expression of a key efferocytosis receptor (MerTK) (116). NPs containing small interfering RNA targeting a macrophage molecule (CaMKIIy) can improve efferocytosis, decrease necrotic core area, and increase fibrous cap thickness – signs of atherosclerotic plaque stability – in a preclinical model of atherosclerosis (117). Certainly, macrophage-targeted nanomedicine holds unique promise (118). Work from the Leeper laboratory has demonstrated that anti-CD47 (pro-efferocytic therapy), delivered systemically (119, 120) or in a macrophage-specific nanotube therapy (121), holds promise in animal models of atherosclerosis, while human data has suggested anti-CD47 therapy reduces carotid artery inflammation (122). Whether the endothelium can or should be exploited for these delivery methods remains to be determined. Within the sphere of resolution biology, pro-resolving lipid mediators such as the resolvins and maresin, have also shown promise (85, 123, 124). While several papers have focused on the effect these mediators have on macrophage function (125–127), it is notable that endothelial cells release resolvin-D1 as a pro-resolution response to a range of low-density lipoproteins (84). Given the ongoing intercellular communication regulated by the endothelium over the lifetime of an atherosclerotic plaque, it behooves us to determine how ECs govern efferocytosis and resolution of inflammation. Lastly, understanding how the endothelium governs plaque stabilizing features will likewise provide new therapeutic targets. Stable plaque phenotypes can be induced through endothelial-specific targets, with EC-deletion of the P2Y2 receptor (regulates VCAM-1 expression and vascular inflammation) (128) and EC-deletion of CD40 (leukocyte adhesion) (129). MicroRNA have also been implicated, with microRNA-210 and−21 both showing the potential to stabilize fibrous caps in advanced atherosclerotic plaques (130, 131). Lastly, sophisticated work from the Owens laboratory has shown that endothelial cells can undergo EndMT and contribute to atherosclerotic fibrous cap stability as a means to compensate, at least for a time, the role usually played by smooth muscle cells (64). Whether we can use ECs as the “trojan horse” to drive any of these processes through specific EV cargo requires exploration. Importantly, understanding the nature of distinct endothelial crosstalk with cells in both the luminal and abluminal space may yield additional critical insights and new targets.

## Discussion: Challenging Dogma for Novel Insights

As discussed in the preceding paragraphs, ECs are dynamic, they anchor polarized communication hubs (e.g., “hot spots” for leukocyte transmigration) and have the capacity to shift identity and transform their regional ultrastructure. In the context of atherosclerotic plaque progression, where the microenvironment is continuously evolving, the endothelium likewise can provide dynamic responses both locally and systemically. One challenge will be to improve upon insights from single-cell transcriptomics to distinguish between the endothelium within the vessel lumen vs. that within the adventitia or vasa vasorum (58, 132). For this, we will need emerging techniques such as microdissection, spatial transcriptomics (133) and imaging mass spectrometry (134) to provide loco-regional information on endothelial phenotype and function in atherosclerosis. Being able to target the endothelium in a site-specific manner will be a critical advance for any therapeutic target. In this way, NPs that can target ECs for robust genome editing holds promise (135). Ongoing work will be required to better understand the mechanisms of EC-enriched NP or EV uptake, followed by evaluation in large animal models. In this light, it is encouraging that endothelial small interfering RNA delivery in non-human primates can be performed using NPs that target multiple organs (136). EV- or NP-based approaches will ultimately need to target regions of activated endothelium, and in this way, should capitalize on VCAM-1 expression for therapies or molecular imaging (105, 137–139). More recent work on the endothelial transcriptome suggests that epigenetic targets exist for anti-atherogenic therapy such as EZH2 antagonism and SIRT1 agonism – either through epigenetic drugs or epigenetic editing [reviewed in (140)]. If endothelial communication is distinct between the luminal (i.e., circulation) and abluminal (i.e., vascular wall) environments, then it is conceivable that therapies will need to consider how to exploit directional communication. Moreover, with respect to EVs, it will be imperative to improve our rudimentary understanding of EV biogenesis, cargo loading, cellular uptake, and processing (3, 4, 141), done in accordance with standards set by the research field (e.g., MISEV2018 guidelines) (142). Reductionist approaches used to study endothelial function have historically collected supernatants from plate-grown cells. As we look to better understand EC communication in distinct environments, it will be important to determine whether and how mediators are released in a polarized fashion. More sophisticated approaches will need to incorporate other cell types, extracellular matrix proteins, and flow. In this way, we may need to move to organ-on-a-chip methods (143) to better elucidate the ideal endothelial communication strategy we wish to either boost or interrupt. Likewise, more complex animal models will be required to track endothelial communication and target endothelial gene editing accordingly (135, 144). While these suggestions add inherent complexity to our research paradigms, they will serve useful as we strive to identify circulating biomarkers of endothelial health and disease or novel targets that directly affect the endothelium and/or can be conveyed across this strategic layer to target cells below.

## Author Contributions

KH conceived the idea, synthesized the literature, and drafted the review article. MC and JF provided conceptual insight and manuscript editing. All authors have read and approved the published version of the manuscript.

## Funding

KH was supported by a Canadian Institutes of Health Research (CIHR) Project Grant (PJT178006) and the Wylie Scholar Award (Vascular Cures), Blair Early Career Professorship in Vascular Surgery (University of Toronto), Peter Munk Cardiac Centre, and University Health Network. MC holds a Tier I Canada Research Chair and a CIHR Foundation Grant (FDN-154299). JF was supported by project grants from the CIHR (PJT148487 and PJT173489). MC and JF are supported by a team grant from the Medicine by Design program, which received funding from the Canada First Research Excellence Fund. JF received infrastructure funding from the John R. Evans Leaders Fund and the Ontario Research Fund (Canada Foundation for Innovation) and is the recipient of a Tier II Canada Research Chair from CIHR.

## Conflict of Interest

The authors declare that the research was conducted in the absence of any commercial or financial relationships that could be construed as a potential conflict of interest.

## Publisher's Note

All claims expressed in this article are solely those of the authors and do not necessarily represent those of their affiliated organizations, or those of the publisher, the editors and the reviewers. Any product that may be evaluated in this article, or claim that may be made by its manufacturer, is not guaranteed or endorsed by the publisher.

## Abbreviations

CD: cluster of differentiation
EC: endothelial cell
NP: nanoparticle
EndMT: endothelial-to-mesenchymal transition
EV: extracellular vesicle
EZH2: enhancer of zeste homolog 2
SIRT1: sirtuin 1
ICAM-1: intercellular adhesion molecule-1
IL: interleukin
PAR3: partitioning defective 3
KLF2: Kruppel-like factor 2
LDL: low-density lipoprotein
TAZ: tafazzin
VCAM-1: vascular cell adhesion molecule 1
VE-cadherin: vascular endothelial-cadherin
YAP: yes-associated protein
MerTK: MER proto-oncogene tyrosine kinase
CaMKIIy: Ca^2+^/calmodulin-dependent protein kinase II
SR-B1: scavenger receptor class B type 1
FMD: flow-mediated dilatation
TGF: transforming growth factor
TNF: tumor necrosis factor
LDL: low density lipoprotein
EndICLT: endothelial-to-immune cell like transition
MAPK: mitogen-activated protein kinase
ERK1/2: extracellular signal-regulated kinase
HIF-1: hypoxia-inducible factor.
